# Prognostic factors in patients with loco-regionally advanced gastric cancer

**DOI:** 10.1186/s12957-017-1243-z

**Published:** 2017-09-15

**Authors:** Bo Hultman, Ulf Gunnarsson, Peter Nygren, Magnus Sundbom, Bengt Glimelius, Haile Mahteme

**Affiliations:** 1 Department of Surgical Sciences, Uppsala University, SE-751 85 Uppsala, Sweden; 2 Department of Surgical and Perioperative Sciences, Umeå University, SE 901 85 Umeå, Sweden; 3 Department of Immunology, Genetics and Pathology, Uppsala University, SE-751 85 Uppsala, Sweden

**Keywords:** Surgery, Gastric cancer, Peritoneal, Metastases, Prognostic factor, Loco-regionally advanced cancer

## Abstract

**Background:**

The aim of this study was to investigate epidemiologic and prognostic factors relevant to the treatment of loco-regionally advanced gastric cancer (GC).

**Methods:**

Two hundred and fifty-five patients with GC were identified in Uppsala County between 2000 and 2009. Patient records were analyzed for loco-regionally advanced GC defined as tumor with peritoneal involvement, excluding serosal invasion from the primary tumor only, at primary diagnosis or during follow-up. The presence or not of distant metastasis (DM), including hematogenous metastases (e.g., liver, lung, and bone) and/or distant lymph node metastases, was also analyzed. The Cox proportional hazard model was used for multivariate analysis of factors influencing survival.

**Results:**

One hundred and twenty patients (47% of all patients with GC; median age 70.5 years) had loco-regionally advanced disease, corresponding to an incidence of 3.8 per 100,000 person-years. Forty-one percent of these also had DM. Median overall survival (mOS) from the time of the diagnosis of loco-regionally advanced disease was 4.8 months for the total patient cohort, 5.1 months for the subgroup of patients without DM, and 4.7 months for the subgroup with DM. There was no significant difference in mOS between the subgroups with synchronous versus metachronous loco-regionally advanced GC: 4.8 months (range 0.0–67.4) versus 4.7 months (range 0.0–28.3). Using multivariate Cox analysis, positive prognostic factors for survival were good performance status at diagnosis and treatment with palliative chemotherapy and/or radiotherapy. Synchronous DM was a negative prognostic factor. The mOS did not differ when comparing the time period 2000–2004 (5.1 months, range 0–67.4) with the period 2005–2009 (4.0 months, range 0.0–28.3).

**Conclusion:**

Peritoneal involvement occurred in almost half of the patients with GC in this study and was associated with short life expectancy. New treatment strategies are warranted.

## Background

In 1975, gastric cancer (GC) was the most common neoplasm worldwide. Even though its incidence is decreasing, it is still common throughout many regions in the world [1], with the highest incidences in East Asia, Eastern Europe, and South America. GC is currently the second most common cause of death globally (10% of all cancer deaths), and adenocarcinoma constitutes 90% of all gastric malignancies [2]. In comparison, Sweden has a relatively low incidence (12 and 6–7 cases per 100,000 men and women, respectively) [3]. A major reason for the divergence in incidence between regions is the variation in prevalence of *Helicobacter pylori* infection [4].

GC is often diagnosed late, since symptoms usually become obvious at an advanced stage. Advanced GC (stage IV) is present in about 20–30% of patients at diagnosis [5]. Median survival in stage IV is short, and there is no long-term survival [6]. In a recent nationwide Swedish registry study (*n* = 7559), peritoneal metastases (PM) in GC were found in 32% of cases [7]. Young age, location other than cardia, signet cell type, and the number of distant metastases were the risk factors for PM.

In patients with GC undergoing resection with curative intent, 10–20% have PM [8], and in an autopsy series, 50% of patients with GC had PM [9]. Advanced GC is mostly treated with palliative chemotherapy, with a median overall survival (mOS) of 7–10 months in recent clinical trials [10, 11]. In cases treated with palliative resection, a mOS of 7–8 months has been observed [12, 13], compared to patients treated with palliative chemotherapy with a tendency of higher mOS.

In non-trial patients or in patients not actively treated, it is considerably shorter [12].

In patients with advanced GC, there is still a lack of data on incidence, prognosis, treatment, and outcome in the subgroup with PM. A recent retrospective analysis of a rather small Japanese material (*n* = 79) failed to identify any prognostic factor other than N3 disease [14]. In that study, patients treated with curative resection and chemotherapy had a mOS of 22 months compared to 10 months for those who had chemotherapy alone.

In recent years, hyperthermic intraperitoneal chemo (HIPEC) therapy has been increasingly used for neoadjuvant treatment of PM in GC. As yet, only small series have been published, but one review has indicated a positive effect on mOS, though not on long-term survival [15]. An interesting development is the use of neoadjuvant *laparoscopic* HIPEC aimed to reduce the extent of PM expressed as the peritoneal cancer index (PCI), thus increasing the proportion of patients eligible for curative resection [16]. In the development of new treatment modalities, a better knowledge of prognostic factors, apart from the widely used PCI score, is needed.

The aim of this study was to investigate epidemiologic and prognostic factors in patients with loco-regionally advanced GC defined as tumor with peritoneal involvement, excluding serosal invasion from the primary tumor only, as well as analyzing patients with or without distant metastasis (DM).

## Methods

GC was defined as an adenocarcinoma with the major tumor volume in the stomach. The International Union Against Cancer system for the classification of malignant tumors, version TNM6, was used for staging. Patients with GC defined in this way and diagnosed in Uppsala County between January 1, 2000, and December 31, 2009, were identified from the Uppsala University Hospital database. The total patient cohort was matched against two registries at the National Board of Health and Welfare: the Swedish Cancer Registry and the Cause of Death Registry. Patient records from all identified cases were assessed for the presence of loco-regionally advanced GC, defined as tumor with peritoneal involvement, excluding serosal invasion from the primary tumor only, at diagnosis or during follow-up, as well as the presence or not of distant metastasis (DM), defined as hematogenous and/or distant lymph node metastases. Demographic data, histopathologic data, and data on symptoms, treatment, and mOS were also extracted. The Regional Ethics Committee approved the study for data extraction during the time period 2000–2009 (Dnr 2007/364).

Median overall survival (mOS) was defined as the median time from diagnosis of loco-regionally advanced GC until death. To determine the possible impact of time-related changes in staging and treatment, patients from two time periods, 2000–2004 and 2005–2009, were analyzed separately. Patients were characterized according to the histopathologic data, synchronous or metachronous disease, and whether or not palliative treatment (e.g., chemotherapy and/or radiotherapy) had been given.

Patients were also classified according to age (above/below 70 years) and Karnofsky performance status [17] (KPS 100, 90, or ≤ 80) at the time of the diagnosis of loco-regionally advanced GC. A diagnosis of GC was derived from pathology specimen reports, except in two cases (based on clinical information).

### Statistical methods

mOS is presented as median values and range. Proportional hazard (Cox) regression, Kaplan-Meier, and the log-rank test were used for analyses of factors possibly influencing survival. Comparison between groups was made using the Mann-Whitney *U* test. *P* values less than 0.05 were considered significantly different. The computer software package STATISTICA AXA version 10.0, StatSoft Scandinavia, Sweden, was used for statistical calculations.

## Results

### Incidence and patient characteristics

**Fig. 1:**
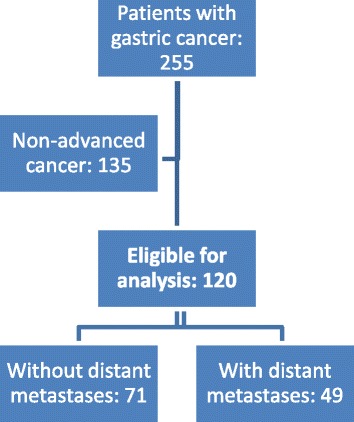
Flow chart depicting the selection process for all patients diagnosed (synchronous and metachronous) with loco-regionally advanced gastric cancer, defined as tumor with peritoneal involvement, excluding serosal invasion from the primary tumor only

Of 255 patients with GC, 120 (47%) fulfilled our criteria for loco-regionally advanced GC and were thus eligible for detailed analyses. The population of Uppsala County in 2000 was 294,196 and in 2009, 331,898, with a period mean of 313,047. The calculated incidence of loco-regionally advanced GC was 3.8 per 100,000 person-years. Loco-regionally advanced disease was synchronous in 80 patients (67%) and metachronous in the remaining 40 patients (33%). Diagnosis of loco-regionally advanced GC was verified by histopathology (63 patients, 52.5%), by assessment at surgery (18 patients, 15%), or by radiology (39 patients, 32.5%). Figure 1 presents the flow chart of the selection process.

The median age of the 120 patients with loco-regionally advanced GC was 70.5 years (range 26–91), males had a slight majority (54%), and most patients had a good performance status (KPS 90 or higher in 65%). Table 1 summarizes demographic, basic clinical, and histopathologic data. Seventy-one patients (59%) did not develop DM (synchronous or metachronous) whereas the remaining 49 (41%) did: 34 synchronous and 15 metachronous (see Table 2).

**Table 1:** Demographic data, histologic, basic clinical data, and treatments of the 120 patients at the time of diagnosis of loco-regionally advanced gastric cancer. Percentages in the total column refer to the total number of patients included (*n* = 120)

Variables	2000–2004	2005–2009	Total
Number of patients included	73	47	120 (100%)
Median age in years (range)	71 (26–91)	68 (41–90)	70.5 (26–91)
Gender			
Female	34	21	55 (46%)
Male	39	26	65 (54%)
Karnofsky performance status			
100	32	20	52 (43%)
90	13	13	26 (22%)
10–80	28	14	42 (35%)
Loco-regionally advanced cancer			
Synchronous	47	33	80 (67%)
Metachronous	26	14	40 (33%)
Morphological type			
Signet ring cells (SRS)	18	19	37 (31%)
Adenocarcinoma without SRS	48	27	75 (63%)
Linitis plastica	1	0	1 (1%)
Missing data	6	1	7 (6%)
Differentiation			
Poorly differentiated	48	36	84 (70%)
Moderately differentiated	13	4	17 (14%)
Well-differentiated	1	1	2 (2%)
Missing data	11	6	17 (12%)
Lauren’s classification			
Intestinal type	17	14	31 (26%)
Gastric type	1	1	2 (2%)
Diffuse type	22	12	34 (28%)
Mixed type	0	2	2 (2%)
Missing data	33	18	51 (43%)
Distant metastasis			
No	45	26	71 (59%)
Yes	28	21	49 (41%)
Synchronous	18	16	34 (28%)
Metachronous	10	5	15 (12%)
Palliative treatment			
Chemotherapy	33	20	53 (44%)
Radiotherapy	0	2	2 (2%)
Chemoradiotherapy	5	2	7 (6%)

**Table 2:** Distant metastases in 120 patients with loco-regionally advanced gastric cancer

Variables	Synchronous	Metachronous
Total number of patients	80 (67%)	40 (33%)
Any distant metastases	34 (48%)	15 (31%)
Liver metastases	6 (8%)	6 (12%)
Para-aortic metastases	13 (18%)	5 (10%)
Lung metastases	1 (1%)	2 (4%)
Skeletal metastases	2 (1%)	0 (0%)
More than one metastatic site	12 (17%)	2 (4%)

### Survival and prognostic factors

**Fig. 2:**
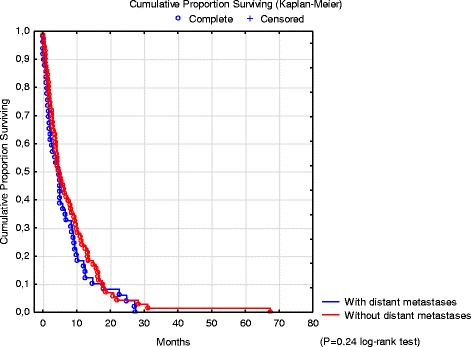
Overall survival of patients with loco-regionally advanced gastric cancer according to the presence or not of distant metastases

**Fig. 3:**
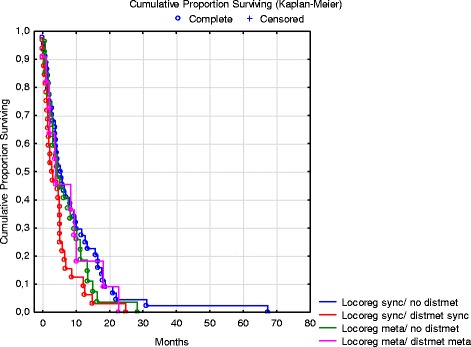
Overall survival in subgroups with synchronous or metachronous loco-regionally advanced gastric cancer according to the presence or not of distant metastases

The mOS of the loco-regionally advanced GC patients was 4.8 months (range 0.0–67.4). In the subgroup of 71 patients without DM, the mOS was 5.1 months (range 0.0–67.4) and in the subgroup of 49 patients with DM, 4.7 months (range 0.0–27.5; Fig. 2). There was no significant difference in OS between the subgroups with synchronous and those with metachronous loco-regionally advanced GC, 4.8 (range 0.0–67.4) versus 4.7 months (range 0.0–28.3). For details on mOS in the different subgroups, see Figs. 2 and 3. There was no statistically significant difference in mOS between patients with a diagnosis of loco-regionally advanced GC during the time period 2000–2004 (5.1 months, range 0–67.4) and those diagnosed 2005–2009 (4.0 months, range 0–28.3). Corresponding mOS for patients treated with palliative chemotherapy was 8.6 months (range 2.7–67.4) and 6.9 months (range 1.2–28.3), respectively. These groups were similar with respect to risk factors (see Table 1).

### Multivariate Cox analyses

Good performance status (Karnofsky > 80) at diagnosis and palliative chemotherapy (alone or with palliative radiotherapy) were associated with a longer OS. The major negative prognostic variable was synchronous DM. Due to a small number of patients, the subgroup’s well-differentiated tumor, grade according to Lauren’s classification (intestinal or diffuse type), metachronous loco-regionally advanced GC with synchronous DM, and patients having palliative radiotherapy were not included in the analyses. Table 3 summarizes the details of the Cox regression analyses.

**Table 3:**
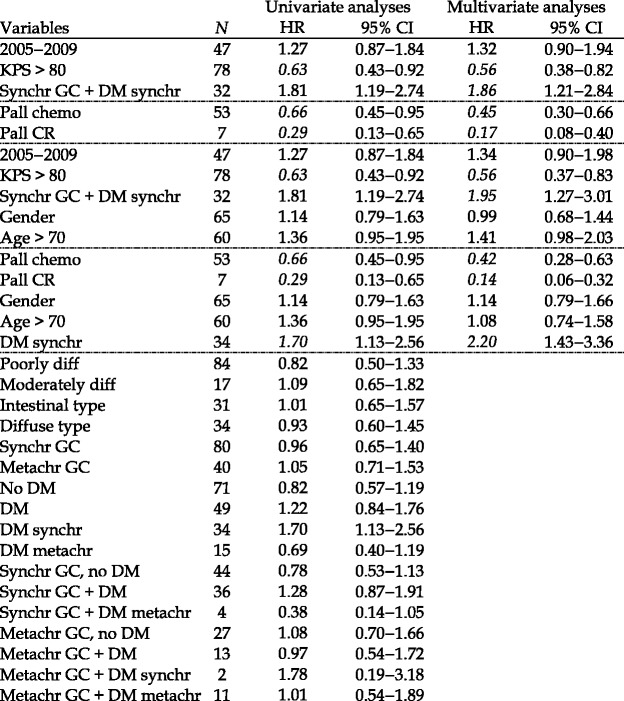
Uni- and multivariate proportional hazard (Cox) regression for overall survival from 120 patients with loco-regionally advanced gastric cancer (GC)

Multivariate analyses were performed in four groups between the dotted lines. Hazard ratios with 95% CI not including the ones in italics

*KPS* Karnofsky performance status, *DM* distant metastases, *Pall* palliative, *CR* chemoradiotherapy

## Discussion

In previous studies, 20 to 32% of GC cases have been loco-regionally advanced. These studies, however, have not specifically looked at these patients, making it difficult to compare their data with ours. Karpeh et al. [18] found PM at diagnostic laparoscopy in ten (20%) out of 50 patients with GC or adenocarcinoma in the esophagogastric junction, all judged as M0 on a clinical basis. A Chinese study detected PM in 24% (33/135) of proximal GC patients after curative resection [19]. In a recent large Swedish multi-registry study without survey of patient records, PM was reported in 32% of cases [7]. Our finding of a 47% PM rate in GC patients is more in line with an autopsy series where PM was detected in 50% of patients with GC [9]. However, it is reasonable to presume that some cases of PM went undiagnosed in our study population, where few autopsies were performed and assessment of PM within the framework of routine treatment and care. In the current study, GC with PM was discovered during follow-up in 33% of patients, a result well in line with the literature [20, 21].

Prognostic factors for GC in general are quite well established but less so in patients with PM with or without DM. Synchronous DM was confirmed as a major independent negative prognostic factor in multivariate analysis, and the mOS in this subgroup of 34 patients was 3.7 months (range 0–25), comparable to 3 months in a French study [22]. Involvement of the liver and skeleton is associated with poor prognosis, with a mOS of 2 months [7]. Good performance status at diagnosis and palliative chemotherapy were found to be major independent positive prognostic factors, a result well supported in the literature [7, 23–26]. In an attempt to study malnutrition and sarcopenia as prognostic factors, we added nutritional and anemia parameters in the multivariate analyses; however, our retrospective data was insufficient to find any differences between patients with and without loco-regionally advanced GC.

The clinical debut of a new generation of chemotherapeutic drugs at the time of this study could, to some extent, explain the 2 months longer mOS compared to that reported by Sadeghi et al. 2000 [22]. The considerably longer mOS observed in recent trials on new drugs for advanced GC [10, 11], compared to the current study, is most likely due to the use of improved oncological regimes, although patients were somewhat younger and had a better performance status than patients in our study. In two old [12, 13] (mOS of 5.4–5.6) and one recent study where some patients also received palliative chemotherapy (mOS 10 months) [14], the mOS in the group of patients without resection was similar to that in the current study. However, the recent REGATTA trial concluded that gastrectomy followed by chemotherapy does not improve survival in advanced gastric cancer, when compared with chemotherapy alone [27].

A recent Japanese study indicated N3 disease to be the only significant negative prognostic factor in GC patients with PM [17]. An American study group [28] found that positive cytology, i.e., stage IV disease according to the 7th edition of the American Joint Committee on Cancer Staging [29], was the most negative preoperative prognostic factor. The mOS in patients with positive cytology undergoing microscopically radical gastrectomy was 15 months, versus 98 months for the group of patients with negative cytology. Mezhir et al. [30] revealed that there was no difference in the mOS between a group of patients with positive cytology undergoing resection and a group without resection. A mOS of 12 months with no patient surviving 3 years was reported by Gold et al. [31] in positive cytology patients treated with neoadjuvant chemotherapy followed by gastrectomy. Based on these results, multimodal therapy, i.e., bi-directional chemotherapy [32, 33] or cytoreductive surgery and HIPEC [7, 34], seems to be a potentially beneficial treatment option. A further refinement of HIPEC therapy, recently published, includes preoperative neoadjuvant treatment aimed to reduce peritoneal metastases (i.e., the PCI score) and thus increases the proportion of patients eligible for curative resection [16]. Such developments increase the need for valid prognostic factors in order to be able to select patients that will benefit the most from each form of therapy. One such option, though as yet not systematically evaluated, is the routine assessment of PCI at preoperative laparoscopy.

This study has a number of limitations. It is retrospective, and the number of patients is quite small. However, 100% follow-up was achieved. The accuracy of the diagnosis of loco-regionally advanced GC may have been slightly distorted since judgments were not always based on pathology reports but in some cases on radiological reports alone. Furthermore, we did not have access to detailed data on the type of palliative oncologic treatment given. Strengths of the study are the following: its focus on loco-regionally advanced GC, the fact that completeness was matched against two independent registries at the National Board of Health and Welfare, and the accuracy in identifying loco-regionally advanced GC, with all medical charts and pathology reports being scrutinized.

## Conclusion

We found peritoneal spread from GC to occur in almost half of patients diagnosed with GC and that PM was associated with short life expectancy. The lack of improvement in mOS over the time period studied indicates the need for novel strategies for earlier diagnosis and more effective treatment. In this respect, cytoreductive surgery and HIPEC therapy in combination with systemic chemotherapy may represent a potentially beneficial option for patients with peritoneal spread but not distant metastases.

## Abbreviations

DM: Distant metastasis
GC: Gastric cancer
HIPEC: Hyperthermic intraperitoneal chemo
mOS: Median overall survival
PCI: Peritoneal cancer index
